# Ultrafiltration of natural organic matter from water by vertically aligned carbon nanotube membrane

**DOI:** 10.1186/s40201-015-0207-x

**Published:** 2015-06-05

**Authors:** Ali Jafari, Amir Hossein Mahvi, Simin Nasseri, Alimorad Rashidi, Ramin Nabizadeh, Reza Rezaee

**Affiliations:** Department of Environmental Health Engineering, School of Public Health, Tehran University of Medical Sciences, Tehran, Iran; Center for Water Quality Research (CWQR), Institute for Environmental Research (IER), Tehran University of Medical Sciences, Tehran, Iran; Center for Solid Waste Research (CSWR), Institute for Environmental Research (IER), Tehran University of Medical Sciences, Tehran, Iran; Nanotechnology Research Institute of Petroleum Industry (RIPI), West Blvd. Azadi Sport Complex, Tehran, Iran; Kurdistan Environmental Health Research Center, Kurdistan University of Medical Sciences, Sanandaj, Iran

**Keywords:** Membrane, Carbon nanotube, Fouling, Natural organic matter, Ultrafiltration

## Abstract

In this study vertically aligned carbon nanotubes (VA-CNT) was grown on anodized aluminum oxide (AAO) substrate. The synthesized AAO-CNT membrane was characterized using Raman spectroscopy, field emission scanning electron microscopy (FESEM), contact angle and BET. The pure water flux, humic acid (HA) (as representative of natural organic matters) rejection and fouling mechanism were also evaluated. The fabricated membrane has pore density of 1.3 × 10^10^ pores per cm^2^, average pore size of 20 ± 3 nm and contact angle of 85 ± 8^o^. A significant pure water flux of 3600 ± 100 L/m^2^.h was obtained at 1 bar of pressure by this membrane due to the frictionless structure of CNTs. High contact angle exhibited the hydrophobic property of the membrane. It was revealed that HA is primarily rejected by adsorption in the membrane pores due to hydrophobic interactions with HA. Flux decline occurred rapidly through both cross flow and dead end filtration of the HA. Based on the blocking laws, internal pore constriction is dominant fouling mechanism in which HA adsorbs in membrane pores results in pores blockage and flux decline.

## Introduction

Natural organic matters (NOMs) are known as problematic substances in environment and health. Nowadays their various direct and indirect effects are well understood. They own different characteristics in terms of reactivity, structure and they enter to water bodies through various natural and man-made sources [1]. NOMs are present to different extents in waters [2, 3]. Typical ranges of 0.1–0.2 mg/L and 1–20 mg/L of NOM based on total organic carbon (TOC) have been reported for ground and surface waters, respectively. However, the TOC concentrations can be very higher (100–200 mg/L) in colored waters of swamps and marshes [4, 5].

These substances form hazardous disinfection byproducts (DBPs) such as trihalomethanes (THMs) in reaction with chlorine. NOM is often made of two fractions, namely hydrophobic and hydrophilic. Hydrophobic and hydrophilic fractions have the potential of THMs and haloacetic acids (HAAs) formation, respectively. Hydrophobic components are humic acid and fulvic acid and the hydrophilic components are proteins, amino acids and carbohydrates [4, 5].

Hydrophobic compounds have the greatest effect on DBPs formation. Furthermore, some hydrophobic substances may intrinsically be much more toxic than the chlorinated components [6].

The presence of NOMs have other problems such as negative effect on the water quality and treatment process, increasing the coagulants and disinfectants demand, biological growth in distribution network, decomposition of organic matter within the network and creating a slimy layer on the pipes [5, 7]. In addition, NOM adversely affects the membrane performance in water purification [8].

Different methods with varying efficiencies such as chemical coagulation and precipitation, ion exchange, adsorption, electrocoagulation, advanced oxidation and membrane process have been applied for NOM mitigation [5, 9–14]. Although in some cases enhanced coagulation has been proposed for NOMs removal, but large application of coagulants, pH modification problems and large amount of produced sludge are the main related obstacles of this method. Furthermore, adsorption as a known process in water treatment, have been studied and applied for NOM removal using various materials. In addition to activated carbon materials, new nano adsorbents such as CNTs and zero valent iron nanoparticles have also been studied as promising adsorbents [15, 16], but the questions related to release of nanomaterials and regeneration costs are the main drawback of adsorption application.

Membranes are used for NOMs fractions removal from aqueous solution as one of the important processes in water treatment. Membrane process is of interest due to no changes in the structure of pollutants, no intermediates addition to water, no adverse environmental effects, no need of chemicals and easy navigation [17].

The major obstacles associated with the membrane are energy consuming and fouling problem. Researchers are attempting to change the structure of conventional polymeric membranes or developed new membranes with higher permeability and higher pollutant rejection.

In this regard, carbon nanotubes (CNTs) have been considered for membrane synthesis due to their exceptional properties and high adsorption capabilities [18, 19]. Due to porous structure of the tubes and high surface area, a wide range of contaminants have been effectively removed by CNTs [20].

Promising results show that CNTs in membranes structure have higher flux, higher performance, less fouling, less required cleaning, higher thermal stability, higher consistency and lower energy requirement than conventional polymeric membranes [21–23].

Carbon nanotube membranes can be synthesized by various methods. One of these techniques is template carbonization to synthesize CNTs with desired diameter and high purity. In this method, the situation is prepared in such a way that the CNTs grow inside the channels of anodic aluminum oxide (AAO) template [23]. Thus, the membrane is created through growing CNT arrays and known as vertically aligned carbon nanotubes (VA-CNT) membrane.

One of the most important advantages of the VA-CNT membrane is high flux of water through the CNTs due to low length to the high density of nanotubes [21]. The first plan of VA-CNT membranes was developed by Hinds research team that CNTs were grown on iron as a catalyst using chemical vapor deposition (CVD) method [21]. It has been reported that hydrophobic channel of CNT is smooth and frictionless that facilitate the rapid movement of water [21]. Accordingly, in recent years, CNTs have been mixed with polymers in order to improve the performance of polymeric membranes with higher flux and less fouling [24–29].

To our knowledge, relatively few studies have been conducted in the development of vertically aligned CNT membranes for water purification. With regard to promising results related to CNTs application for membrane synthesis, present work is going to synthesize and characterize the VA-CNT membrane through AAO technique and investigate for NOMs rejection from water.

## Materials and methods

### Membrane experimental set up

**Fig. 1:**
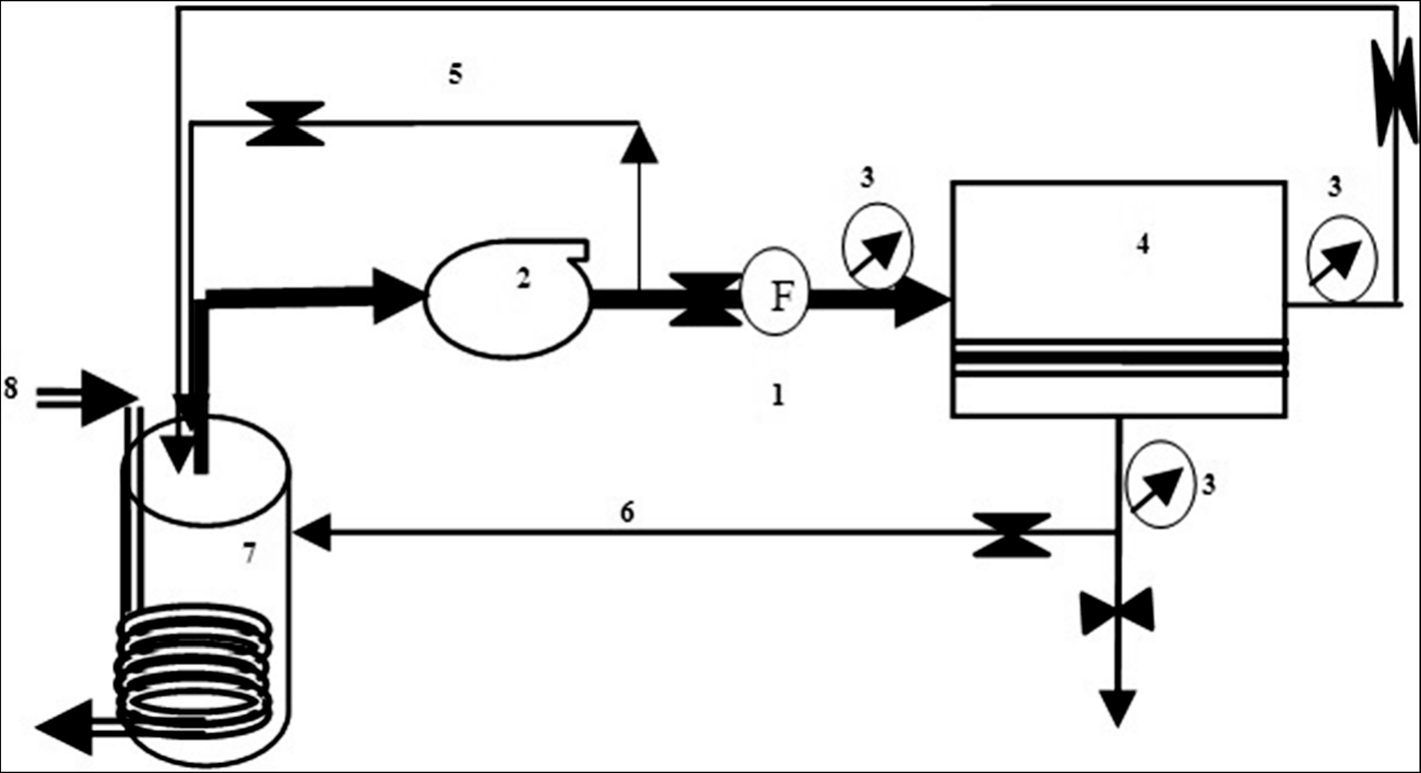
Simplified schematic of experimental set-up unit. 1. Flow meter 2. Low pressure pump 3. Gauges 4. Membrane module 5. Recirculation (bypass) line 6. Concentrate line 7. Feed tank 8. Cooling circuit

The schematic diagram of the experimental setup is shown in Fig. 1. It consists of a 2-L feed tank, membrane module with the effective area of 1.4 cm^2^, feeding pump, cooling system, valves, gauges and flow meters.

### Synthesis, preparation and characterization of membrane

All the chemicals reagents were of reagent grade and no further purification was done. The fabricated membrane was synthesized via anodic aluminum oxide (AAO) method through a two-step process (anodizing and growth of CNTs in the porous AAO).

In this study, a similar procedure as Gilani et al. was used for membrane synthesis and preparation [22, 30].

High purity (99.99) aluminum (Al) foil of 300 μm thickness was cut in small rectangular pieces. The foils were sonicated in acetone solution and then rinsed with double distilled water in order to degrease and subsequently dried in room temperature. Then, Al plates were electropolished in ethanol and perchloric acid (60 %) mixture solution (4/1 v/v) under constant cell voltage of 20 V for 2 min. The back surface of the plates was protected by an insulting tape.

The anodizing process started using oxalate acid (0.3 M) as electrolyte solution and under voltage of 40 volts for 2 h. Separation of the oxide layer was conducted using a mixture of phosphoric acid (6 % wt) and chromic acid (1.8 wt %) for 2 h at 65 °C.

Aluminum foil was re-anodized for 58 h under identical manner used for first anodization step. Then the unoxidized part was removed by putting in saturated mercuric chloride. The barrier layer was removed by soaking the template in phosphoric acid (5 wt %) at temperatures of 50 °C for 3 h [22, 30]. Deposition of CNTs onto the interior walls of the template was conducted by placing the AAO in CVD furnace. The temperature of the furnace was gradually increased to 650 °C at a rate of 5 °C/min. Meanwhile, a controlled argon flow (200 ml/min) was induced to the furnace. A mixture of acetylene and argon was inserted into the furnace as the carbon precursor and carrier gas, respectively with a ratio of 0.01 for 12 h. After the carbon deposition, the acetylene flow was turned off, under the condition of argon flow the reactor was allowed to cool to room temperature for 12 h. Finally, the synthesized membrane was washed in ethanol and dried in vacuum oven at 60 °C [22, 30].

Membrane characteristics were performed using Raman spectroscopy, FESEM, contact angle, and BET. Field emission scanning electron microscopy (FESEM) (Hitachi-S4160) was used to characterize the uniformity and morphology of the AAO–CNT membrane. The specific surface area of the AAO–CNTs was determined using the Brunauer–Emmett–Teller (BET) method by ASAP 2010 (Micromertics Inc., Norcross, GA).

Raman spectrum was used to observe the uniformity of graphite stracture of the growing CNTs in the membrane using a Raman spectrometer (Almega Thermo Nicolet Dispersive). Contact angle as an important factor in membrane characterization was measured by a contact angle analyzer (OCA 15 plus, dataphysics Instruments, Germany) using the sessile drop technique.

### Solutions and analytical measurements

A laboratory grade humic acid (Acros Organics Company, NJ – USA) was used in this study as NOM model to evaluate the performance and removal mechanism by synthesized VA-CNT-AAO membrane. A known amount of HA powder was dissolved in distilled water and pH was adjusted around 7 for all experiments. The concentration of HA was reported as the term of TOC, as a surrogate measure using a TOC analyzer (TOC-VCPH, Shimadzu, Japan). For this purpose different samples (feed, permeate and concentrate) were taken at defined interval times and analyzed for TOC concentration.

Generally, in membrane process, materials may be removed from water by adsorption and/or repulsion mechanisms. The portion of desorbed or adsorbed solutes on the membrane surface is important in fouling and flux analysis.

From a practical standpoint, to investigate the removal mechanism, a certain volume of synthetic solution was placed in the feed tank (Fig. 1). Then, the system was operated in closed loop condition where permeate and concentrate were recycled back to the feed tank at constant transmembrane pressure (TMP) of 1 bar. A cooling circuit was applied to feed tank to maintain the temperature of the feed solution at a constant value of 25 ± 0.5 °C. Sampling was performed at specified intervals from feed tank, permeate and retentate flows for further analysis. All experiments were conducted in duplicate. The observed percentage of HA rejection (%) was calculated as Eq. 1.

1 $$ \%R=\left(\frac{Cf-Cp}{Cf}\right)\times 100 $$

Where C_f_ and C_p_ are TOC concentrations in feed solution and permeate, respectively.

The adsorbed mass of TOC is equal to initial TOC mass in the feed tank minus the sum of TOC mass in the feed tank and cumulative samples of permeate at the end of experiment period, as determined by following mass balance equation (Eq. 2).

2 $$ A. Mads= CfiVfi-\left[ CffVff+{\displaystyle \sum CpVp}\right] $$

Where *A* is the effective membrane area. *M*_*ads*_ is the adsorbed mass of TOC per surface area of membrane. *C*_*fi*_, *C*_*ff*_ and C*p* are the initial concentration of TOC, final concentration of TOC in the feed tank and concentration of TOC in permeate respectively. *V*_*fi*_, *V*_*ff*_ and *V*_*p*_ are the initial volume of feed solution in the tank, final volume of solution in the tank and volume of permeate, respectively.

## Results and discussion

### Membrane characteristics

**Fig. 2:**
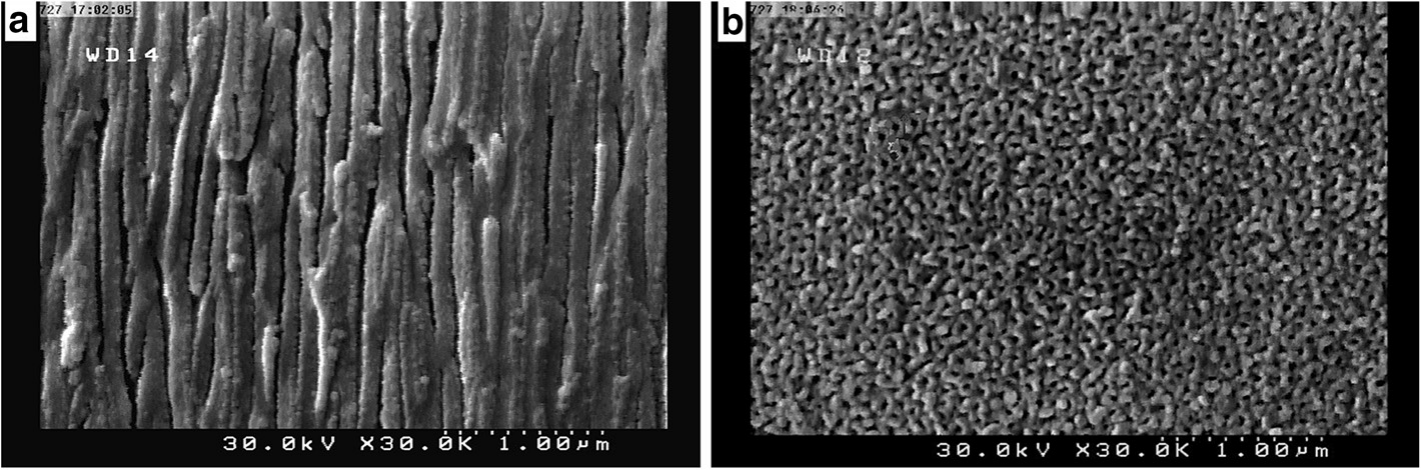
FESEM image of AAO-CNT membrane. **a** cross section of the membrane and **b** surface area image of the membrane

Figures 2a and b illustrate the cross section and surface FESEM image of the membrane respectively. A through hole membrane is clearly shown in Fig. 2a. This image also depicts the direction of cavities and grown CNTs in the holes. The anodizing process and operation conditions (namely voltage, electrolyte concentration and time) effectively influence the properties of the channels [31].

The straight form of the channels indicates the well ordering of CNTs in this fabricated membrane. Fig. 2b shows the pores of the synthesized membrane (dark points). From analysis of FESEM image using the ImageJ analyzer [32] a highly uniform distribution of pores arrangement and nearly uniform pore size (≈20 nm) was created which is in the range of a UF membrane. As seen in Fig. 2b, the surface of the membrane looks uneven. Appling low voltages for AAO synthesis usually results in lower pore sizes and higher pore densities. This property has been shown in other similar works [22, 30]. Therefore, in low voltages due to higher spaces between the pores, the surface is rougher. Cleaning and polishing the surface of aluminum foil can relatively decrease the total surface roughness, but during the anodizing process and growing the CNTs near the top surface, the roughness increases. Although such a roughness may be defined as an advantage for some applications [31] due to higher surface area as an important factor in adsorption process, this can not be of interest for water purification. In particular, high roughness affects the membrane performance in water purification that results in fouling problem.

**Fig. 3:**
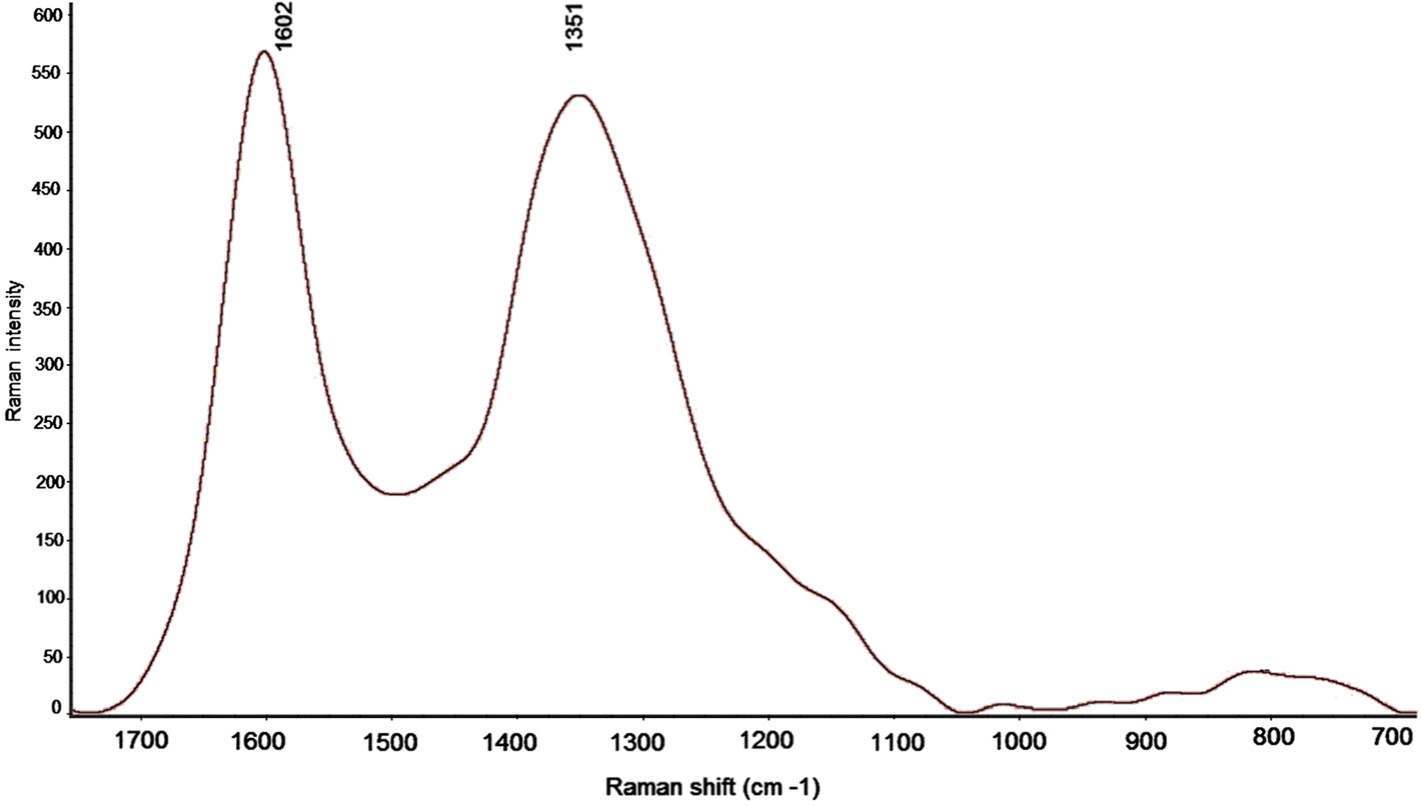
Raman spectrum for AAO-CNT membrane

As depicted in Fig. 3, two strong peaks appeared in the Raman spectrum. The first peak is at around 1351 cm^-1^ (disordered D line band) and the next peak is about 1602 cm^-1^ (ordered G band). From the shift of the Raman spectrum peaks (D and G) the ratio of their intensities (IG/ID) is about one which shows the good structure of CNTs formed in the porous AAO and free from amorphous carbon which is consistent with other researchers [22, 33]. Compare to CNTs grown by other methods, commonly growing on metal cores as catalyst, growing of CNTs in AAO template produce higher purity CNTs with less amporph carbons. Accordingly the need of further purification is not necessary for AAO-CNTs. Nevertheless, this technique produces nearly uniform CNTs in the membrane structure.

**Fig. 4:**
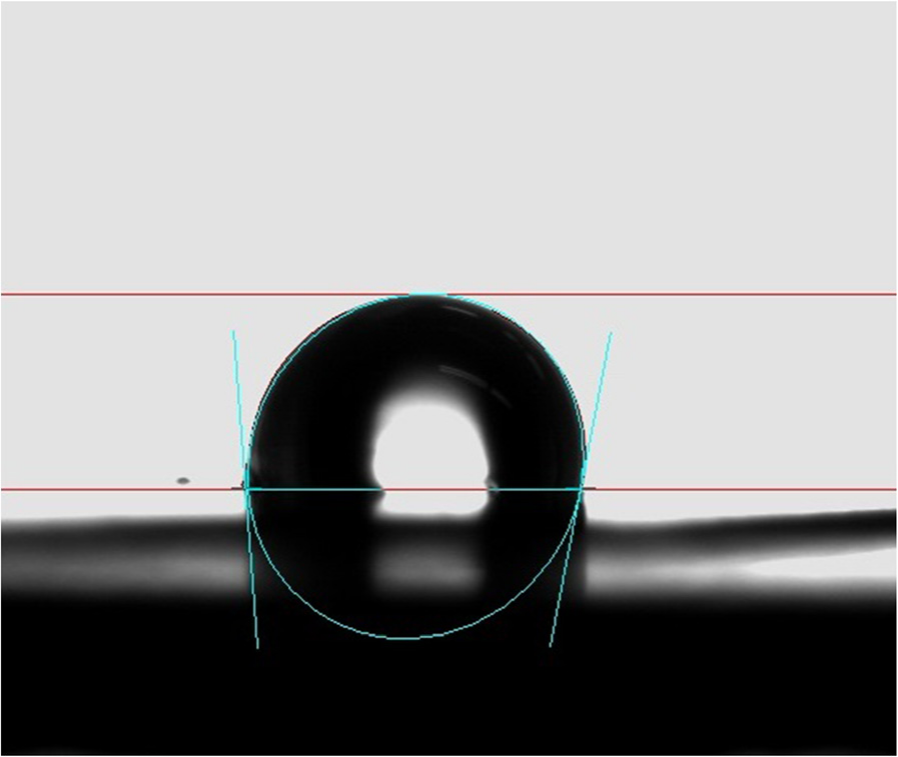
Contact angle image for AAO-CNT membrane surface

Contact angles of several droplets on the surface of the membrane were measured and the average was reported (Table 1). Results showed that AAO-CNT membrane has a hydrophobic behavior as the result of high contact angle. Fig. 4 shows the droplet of pure water on the surface of the membrane and the contact to the surface. Generally, grown VA-CNTs on different substrates may somewhat change the hydrophilicity behavior and contact angle of the VA-CNT membrane due to surface chemical structure and method of synthesis [34]. Since CNTs intrinsically have hydrophobic properties, it has been revealed that membranes fabricated by unmodified or non-functionalized VA-CNTs have close contact angles and are primarily hydrophobe (Table 1). Functionalization of VA-CNT membrane can change the membrane properties and introduce function groups on the CNTs [30].

**Table 1:** VA-CNT membrane characteristics compared to other related works

	This work	Youngbin Baek. et al [37]	Seung-Min Park. et al [38]
Materials	CNT-AAO	CNT-epoxy	CNT-epoxy
Pore density (pore/cm^2^) *10^10^	1.3	6.8	6.8
Average pore size (nm)	20 ± 3	4.8 ± 0.9	4.87 ± 0.87
Pore volume (cm^3^/g)	0.425	Nd^a^	Nd
BET surface area (m^2^/g)	220.4	Nd	Nd
Contact angle (degree)	85 ± 8	74.6 ± 2.8	92.1
Porosity	0.18 ± 0.4	Nd	Nd
Thickness (μ)	~120 ± 20	~200	200 ± 50
Flux(L/m^2^.h) at 1 bar	3600 ± 100	1100 ± 130	1000 ± 100

^a^Not defined

### Permeation analysis

Pure water flux, (J_0_) was calculated using the following equation (Eq. 3):

3 $$ {\mathrm{J}}_0 = \mathrm{V}/\mathrm{A}\mathrm{t} $$

where *V* is the total volume of permeated pure water, *A* is the effective membrane area, and *t* is the operation time.

The pure water permeability for the VA-CNT membrane was 3600 ± 100 L/m^2^.h at TMP of 1 bar. This value is several times than conventional UF membranes for water process. A high practical flux of a commercial UF membrane was reported to be 800 L/m^2^.h-bar flux [35]. Furthermore, due to frictionless structure of VA- CNT channels, it has been reported that fluid flow can be 4–5 times faster than conventional flows [36]. This dramatic velocity can facilitate the application of CNTs channel for other fields.

However, the flux of VA-CNT is significantly higher than that of similar polymeric membranes. In spite of lower pore density, lower pore diameter and higher thickness of aligned CNT membranes, a higher flux (3–4 orders of magnitude) than polymeric UF membrane have been reported [37, 38]. Besides pore size different parameters such as membrane thickness, method of preparation, size distribution can influence the flux of VA-CNT membranes that should be considered for comparing membrane permeabilities. Higher pore diameter (~20 nm) and lower thickness of the membrane result in higher flux in this study. In spite of low pore number of the synthesized membrane a high flux was observed due to lower thickness and also a guarantee of vertically aligned CNTs standing. As shown in Fig. 2a, aligned structure of CNTs in AAO template can facilitate transport of water through the membrane. The number of pores per unit of membrane surface can also affect the membrane flux significantly in combined with other parameters (e.g. membrane structure, thickness and pore number) [21].

### TOC Removal mechanism

**Fig. 5:**
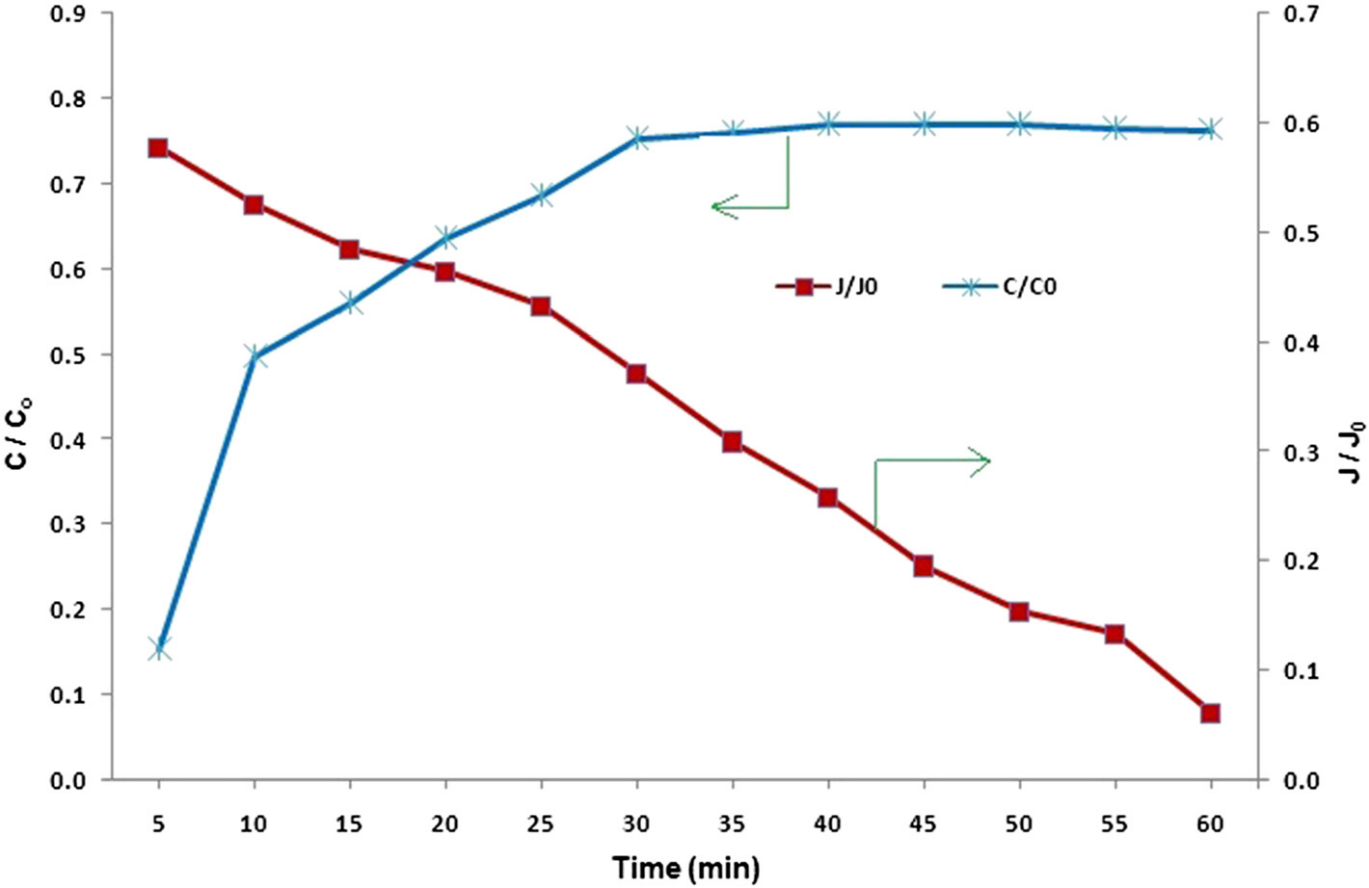
Trend of TOC concentrations in permeate and flux in cross flow operation for initial TOC of 5 ± 0.3 mg/L, pH of 7.0 ± 0.2 at TMP of 1 bar

In cross flow filtration of HA, flux declined after a short period of filtration (Fig. 5). The flux declined to near 0.6 of initial flux (J_0_) after 5 min of filtration (40 % of flux decline). The flux decrease continues to more than 90 % of J_0_ after 60 min of filtration. Interaction of hydrophobic surface of membrane with HA results in plugging of the pores and consequently rapid flux decrease [38]. Principally, adsorption of NOM by CNT is a rapid process and the adsorption capacity is quickly exhausted. From this study, using mass balance equation (Eq. 1), 61 % of the loaded TOC was adsorbed on the VA-AAO-CNT membrane and only 11 % was repulsed. It was also calculated that 30 ± 0.5 g TOC was adsorbed per square meter of the membrane at a constant TMP of 1 bar for 60 min of filtration. Although adsorption may be an effective rejection mechanism during the initial steps of AAO-CNT membrane operation, but due to quick flux decline during the process, it is not an efficient mechanism in long-term filtration. However it may be preferred for some biochemical and biomedical applications for retention of toxin from plasma, some trace contaminants such as endocrine disrupting compounds (EDC) bisphenol A or heavy metals [39–41].

Anyway, determination of removal mechanism is very important. Therefore, making a decision about the best mechanism for removing a specific contaminant or whether depends on several factors. In some cases, the superior removal mechanism may be adsorption or repulsion of pollutants by the membrane, so the adsorption or repulsion capacities can be increased by inducing certain functional groups or by imposing certain conditions. It was revealed that functionalized CNTs with carboxylic groups can somewhat decrease the adsorption on the membrane [42]. From this study materials similar with HA substances can effectively be adsorbed on the CNT membrane types.

### Fouling mechanism analysis

Blocking laws as one of the most popular models were applied to characterize the fouling mechanisms in this study. These empirical models were presented by Hermia [43]:

4 $$ \frac{d^2t}{d{v}^2}=\mathrm{k}{\left(\frac{\mathrm{dt}}{\mathrm{dv}}\right)}^{\mathrm{n}} $$

Where *t* is time (s), *V* is volume (L) k is blocking law filtration coefficient (units depends on *n*) and *n* is blocking law filtration exponent (dimensionless) that expressing the fouling regime. Based on the *n* values different modes of fouling namely pore sealing (*n* = 2), internal pore constriction (*n* = 1.5), pore sealing with super position (*n* = 1) and cake filtration (*n* = 0) can be expressed. To determine the mechanism responsible for fouling, the experiment was conducted under dead end condition at constant pressure of 1 bar. At certain intervals, permeate volumes were recorded. Data was tabulated in a spreadsheet for analysis.

Using the Eq. 4 by plotting log d^2^t/dv^2^) versus (dt/dv) the slope of the line is constant value (*n*) that represent the fouling mechanism [44].

**Fig. 6:**
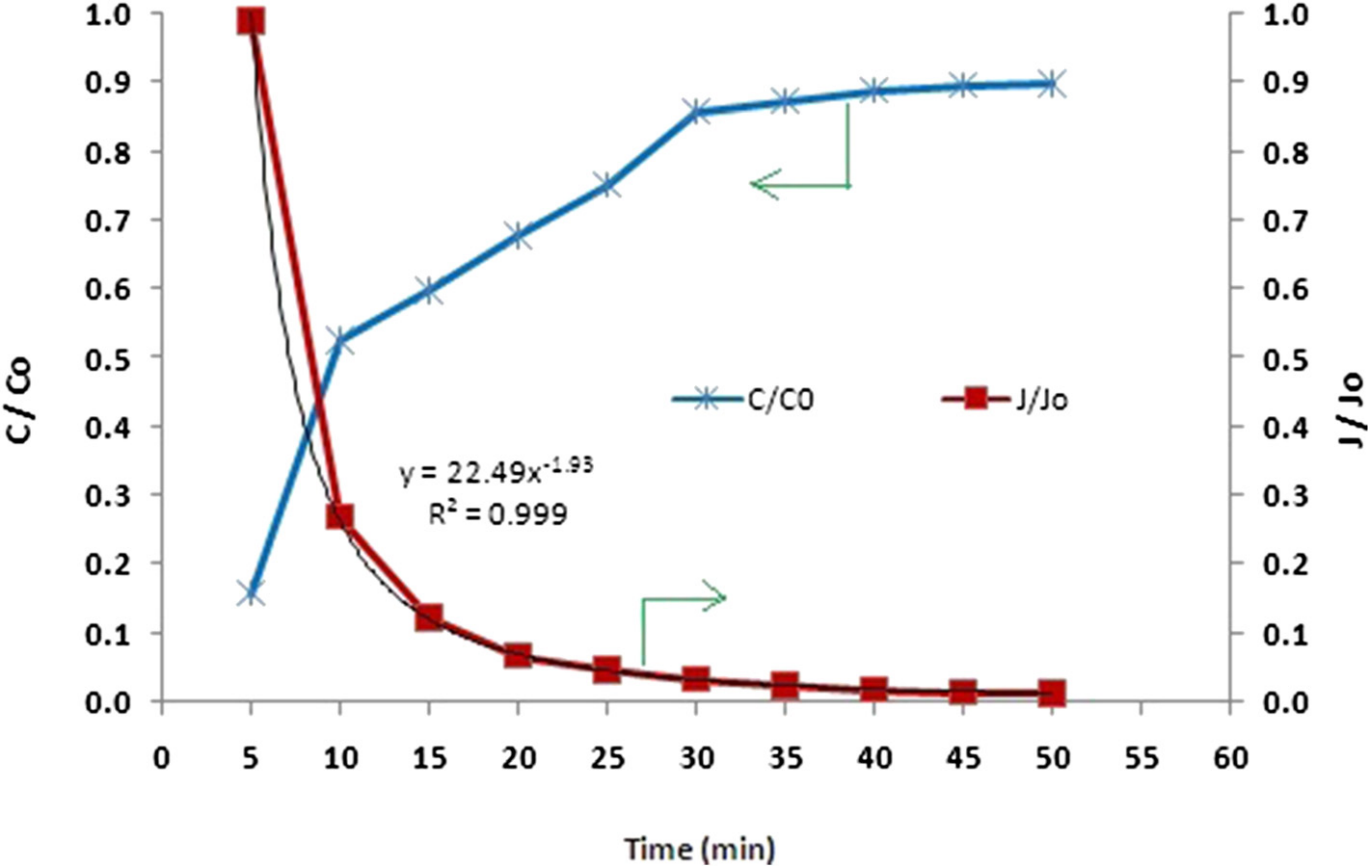
Flux decline and TOC rejection trend in dead end filtration of HA at TMP of 1 bar and initial TOC = 5 ± 0.3 5 mg/L and pH of 7 ± 0.2

In dead end filtration of HA, about 50 % of rejection occurs at first 10 min of operation (Fig. 6). As previously noted, high affinity of CNTs to HA results in rapid adsorption and fast depletion of adsorption capacity. In other word, due to increase in accumulation of HA in the solution, HA penetrate into the membrane, attach to adsorption sites and pass through the membrane. This trend is nearly similar to whatever occurs in the tangential operation, although in cross flow mode the rejection is higher as the operation time elapsing due to the cross flow velocity, in which, some of deposited HA is swept away from the membrane surface.

In this operation mode, the flux declines rapidly (Fig. 6). About 80 % of decline occurs at first 10 min of filtration and after 20 min, it reduce to 90 % that differs from what ever seen in cross flow mode. Increasing of HA in bulk solution due to its accumulation in dead end filtration, result in rapid blocking of the pores and rapid flux decline. Generally, a 90 % of the flux decline occurred at 57 min of filtration in cross flow operation, while this percentage of flux reduction happened after about 15 min of filtration for dead end mode.

**Fig. 7:**
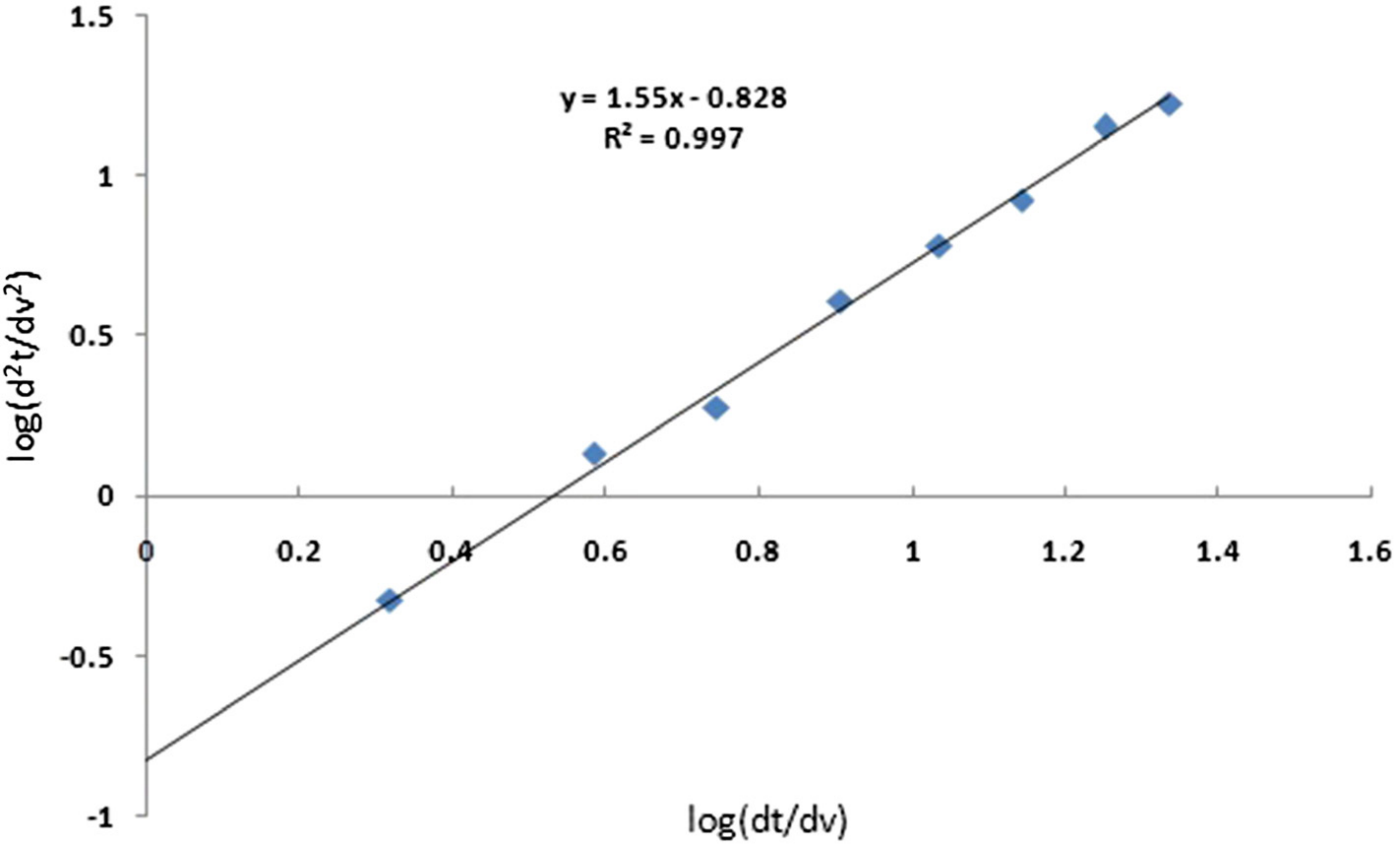
Fouling analysis for HA filtration. Operating conditions: TMP = 1 bar, TOC =5 ± 0.3 mg/ L, pH = 7 ± 0.2

From Fig. 7 based on the equation obtained by plotting the log (d^2^t/dv^2^) versus log (dt/dv) the line slope (*n*) is 1.55. Accordingly, fouling mechanism of the membrane can be expressed by pore constriction or standard blocking filtration law that is defined as the reduction of the cross- sectional area of the membrane pores due to adsorption of HA.

Sufficient small size of the pollutant that penetrate into the membrane and the adsorption affinity of the pollutant to membrane material have been considered as the main factors for pore constriction phenomenon [44]. Primarily rapid adsorption of HA into the membrane pores decrease the pore size and subsequently results in rapid flux decline. Such an adsorption through the membrane pores and channels (Fig. 2a) makes the cleaning difficult and lowers the membrane reversibility to some extent, otherwise, an effective cleaning technique should be developed.

The conditions, mainly membrane characteristics, can reveal different fouling mechanisms [45]. In fouling analysis, a combination of mechanisms may also occur in a filtration process. In some researches, intermediate blocking followed by cake filtration reported under experimental conditions [46]. In general, attempts to reduce the existing fouling by different methods and changing the removal mechanism from adsorption to electrostatic repulsion are of favor to overcome the problem. For existing membrane inducing the negative charges, can be a solution for reduction of fouling.

## Conclusion

Despite of high contact angle and high hydrophobic property of the fabricated CNT-AAO membrane, pure water flux is considerably higher than that of common types of polymeric membranes. Due to high affinity, the membrane rapidly absorbs HA and consequently rapid flux decline occurs because of internal pore constriction as dominant fouling mechanism. It is important to be noted that a challenge related to present fabricated membrane is its frangibility. It is a critical obstacle for larger surface area application and high driven pressures for municipal and industrial applications. However, its application for some laboratory purposes may be beneficial to absorb some materials with the same characteristics as HA. Further studies can focus on methods of the AAO-CNT membranes fabrication with higher tolerability and flexibility for other pollutants removal from water.
